# In Vitro Effect on *Plasmodium falciparum* and In Vivo Effect on *Plasmodium berghei* of Annomaal*,* an Oily Fraction Obtained from the Seeds of *Annona squamosa*

**DOI:** 10.3390/molecules28145472

**Published:** 2023-07-17

**Authors:** Sampada S. Sawant, Satish Y. Gabhe, Kamalinder K. Singh

**Affiliations:** 1 C.U. Shah College of Pharmacy, SNDT Women’s University, Santacruz (West), Mumbai 400049, India; 2 Cipla Limited, Mumbai 400013, India; 3 School of Pharmacy and Biomedical Sciences, University of Central Lancashire, Preston PR1 2HE, UK; 4 Biomedical Evidence Based Transdisciplinary Health Research Institute, University of Central Lancashire, Preston PR1 2HE, UK

**Keywords:** malaria, *Plasmodium falciparum*, *Plasmodium berghei*, *Annona squamosa*, seeds, antimalarial, anti-plasmodial, artemisinin resistance

## Abstract

Malaria remains a life-threatening health problem and is responsible for the high rates of mortality and morbidity in the tropical and subtropical regions of the world. The increasing threat of drug resistance to available artemisinin-based therapy warrants an urgent need to develop new antimalarial drugs that are safer, more effective, and have a novel mode of action. Natural plants are an excellent source of inspiration in searching for a new antimalarial agent. This research reports a systematic investigation for determining the antimalarial potential of the seeds of *A. squamosa*. The study shows that the crude seed extract (CSE), protein, saponin, and the oily fractions of the seeds were nontoxic at a 2000 mg/kg body weight dose when tested in Wistar rats, thus revealing high safety is classified as class 5. The oily fraction, Annomaal, demonstrated pronounced antimalarial activity with low IC_50_ (1.25 ± 0.183 μg/mL) against *P. falciparum* in vitro. The CSE and Annomaal significantly inhibited the growth of *P. berghei* parasites in vivo with 58.47% and 61.11% chemo suppression, respectively, while the standard drug artemether showed chemo suppression of 66.75%. Furthermore, the study demonstrated that oral administration of Annomaal at a daily dose of 250 mg/kg/day for 3 days was adequate to provide a complete cure to the *P. berghei*-infected mice. Annomaal thus holds promise as being patient-compliant due to the shorter treatment schedule, eliminating the need for frequent dosing for extended time periods as required by several synthetic antimalarial drugs. Further studies are needed to determine the active compounds in the oily fraction responsible for antimalarial activity.

## 1. Introduction

Malaria is one of the most serious challenges to modern healthcare. Malaria is caused by five species of parasites of the genus Plasmodium, namely, *P. falciparum*, *P. vivax*, *P. ovale*, *P. malariae,* and *P. knowlesi,* which affect human beings. Malaria due to *P. falciparum* is the deadliest and most predominantly present in Africa while *P. vivax* infection is less deadly but occurs more widely, albeit the three other plasmodium species have less frequent existence [1].

Malaria is a preventable and treatable disease. The global tally of malaria cases reached 247 million in 2021, an increase from 245 million in 2020 and 232 million in 2019 [2]. There were an estimated 619,000 malaria deaths globally in 2021. Over the 2 peak years of the pandemic (2020–2021), COVID-related disruptions led to approximately 13 million more malaria cases and 57,000 more malaria deaths. The WHO African Region continues to report a high share of the global malaria burden. In 2021, the WHO African Region reported approximately 95% of all malaria cases and 96% of deaths. Children under 5 years of age accounted for approximately 80% of all malaria deaths in the region [2].

The best available treatment, particularly for *P. falciparum* malaria, is artemisinin-based combination therapy (ACT). However, the use of oral artemisinin-based monotherapy is considered a major contributing factor to the development and spread of resistance to artemisinin drugs. Artemether lumefantrine (AL) and artesunate amodiaquine (ASAQ) combinations are the first-line treatments recommended by WHO, with ASAQ as a backup in cases where AL is contraindicated [3]. In ACT, artemisinin is a short-acting drug and eliminates the majority of the plasmodium within the first two days of the treatment while the remaining parasites are eradicated by the long-acting partner drug lumefantrine [4], thus preventing the development of drug resistance to the treatment. There are reports of the development of artemisinin resistance in the areas of Southeast Asia [5] and Rwanda [6], which is described as the delayed clearance of the parasite with the presence of more than 10% parasitemia on the third day of the treatment.

New antimalarial drugs that are efficacious in countering the resistant plasmodium strains are therefore necessarily required. Furthermore, many of the current antimalarial drugs have the limitations of poor bioavailability, inadequate efficacy, reports of side effects, and long treatment regimens, which result in poor patient compliance, thus emphasizing the urgency for novel antimalarial drugs.

Key antimalarial drugs such as quinine and artemisinin derivatives have originated from plants [7]. Though there are efforts in this direction, there is still a gap in the documentation and investigation of plants traditionally used for the treatment of malaria [8,9]. Ethnopharmacological investigations of such plants are vital to finding plausible new antimalarial compounds for future antimalarial chemotherapy.

There are reports of species of Annonacea used by herbal practitioners to treat malaria [10,11]. *Annona squamosa* Linn. belonging to the family Annonaceae is native to the West Indies. *A. squamosa* is widely cultivated for its sweet fruit called sitaphal or custard apple. The plant is notably recognized for its distinct therapeutic properties, and the literature reports various investigations into its phytochemical and pharmacological properties [12,13,14]. It is known for its insecticidal, antimicrobial [15,16], and mosquitocidal activities [17]. Antidiabetic [18], cardiotonic [19,20], antioxidant, and anti-lipidimic properties [21] of its leaves have been demonstrated. The anti-tumor activity of the annonaceous acetogenins from the seeds [22], the anti-cancer activity of the leaves and stem extract [23], and the genotoxicity of the methanolic fraction of the seeds [24] have been recorded. Ayurvedic practitioners have used stem and leaf extracts of the plant as an indigenous uterotonic [25]. The seed extract of *A. squamosa* has shown spermicidal [26] and post-coital anti-fertility activity [27]. However, there is no systematic investigation into the antimalarial activity of this plant.

There is one report of the use of a decoction of the leaves of *A. squamosa* to tackle malaria by herbal practitioners of Ewondo and Bamileke tribes in Cameroon, Africa [10]. In another report on the screening of Sudanese medicinal plants for antiplasmodial activity, the methanolic extract of *A. squamosa* leaves and *A. squamosa* stem bark have been shown to possess antiplasmodial activity against the 3D7 and Dd2 *P. falciparum* strains [28]. Singh et al. reported that the leaf ethanol extract of *A. squamosa* showed promising antiplasmodial activity among the 22 plant species assayed from traditional plants used by local healers for malaria in the Chhotanagpur plateau in India [11].

However, there is no report on the antimalarial activity of the seed extract of *A. squamosa*. To the best of our knowledge, this is the first systematic report of the antimalarial activity of the seed extract of *A. squamosa.* This study investigated the phytochemical components of the seeds, wherein the seeds were fractionated into five phytochemical components and tested for their toxicity. The crude seed extract (CSE) along with the five fractions were then tested for their antiplasmodial activity, using both the in vitro Trager Jensen technique for *P. falciparum* [29] and an in vivo evaluation of antimalarial activity against *P. berghei* infection following the protocol of Peter’s four-day suppressive test [30]. The oily fraction labelled Annomaal, which showed the highest antimalarial activity, was further investigated to determine the dose and dosage regimen for its use in antimalarial chemotherapy.

## 2. Results

### 2.1. Evaluation of Phyto-Constituents

The *A. squamosa* powdered seeds exhibited the presence of carbohydrates, alkaloids, fats and oils, proteins, saponins, flavonoids and tannins, and phenolic compounds (Table 1). These results are in conjunction with the previous report of Patel et al. [31].

The seed powder was fractionated into various phytoconstituents (Table 2). Hydroalcoholic crude seed extract (CSE) was prepared by Soxhlet extraction after defatting with petroleum ether. The petroleum ether fraction was evaporated carefully to obtain the oil-rich fraction named Annomaal. The alkaloid fraction was prepared from defatted seed powder on percolation with ammonia (10%) and extracted with methanol and concentrated. The saponin-rich fraction was also acquired from defatted seed powder after extracting it with water followed by n-butanol. The n-butanol soluble fraction yielded the saponin-rich fraction while the water-soluble fraction was rich in tannins. The defatted seed powder was percolated with Tris buffer saline in cold conditions, and the extract obtained was treated with ammonium sulphate to precipitate the proteins, which were dialyzed against cold distilled water to get rid of any impurities and thus acquire the protein-rich fraction.

The seed powder showed a maximum yield of alkaloids (76.0%) followed by oils with a yield of 20.98%. The percent yield for saponin, tannins, and protein fractions was low (Table 2).

### 2.2. In Vitro Erythrocyte Toxicity Test

Hemolysis is a type of acute toxicity assay used to evaluate the hemocompatibility of drugs and to detect the hemolyzation of erythrocytes [33]. All the fractions were tested for in vitro hemolytic activity and Triton X-100 was used as a positive control (100% value). Erythrocytes from rat blood were incubated with the various seed fractions and CSE at the concentration of 250 mg/mL for 1 h. CSE, tannins, and the oily and protein fractions showed insignificant hemolysis, i.e., less than the tolerated 5% threshold (Table 3) indicating no detectable detrimental effect on RBC membranes [34]. The saponin fraction caused limited hemolysis (less than 10%) while the alkaloid-rich fraction showed the maximum erythrocyte-damaging effects with 11.54% hemolysis.

### 2.3. In Vivo Acute Oral Toxicity Studies

An acute oral toxicity study was carried out for CSE and the five fractions in female Wistar rats. The test animals were divided into six groups of six animals each and received a single dose of 2000 mg/kg body weight orally of the test sample (CSE or either of the five fractions) and were observed for changes in body weight (Table 4) and changes in behavior abnormality, feeding habits, locomotor changes, and somatomotor activity (Table 5). The alkaloid-rich-fraction-treated animals showed some weight loss (Table 4) and were less active (Table 5). The animals in all other groups showed a steady increase in body weight with no significant changes in behavior or locomotor activities (Table 4 and Table 5). All the animals remained healthy until the end of the study period. There was no mortality observed in any of the animals in any of the groups until the end of 14 days. The CSE, saponin, tannin, oil, and protein fractions thus showed no toxicity at a dose of 2000 mg/kg and can be classified as category 5.

### 2.4. In Vitro Anti-Plasmodial Efficacy Testing in P. falciparum-Infected RBCs

CSE and all the fractions were evaluated in vitro for their antiplasmodial activity using *P. falciparum* 3D7 parasite-infected RBCs and IC_50_ values were determined (Table 6).

The oily fraction, Annomaal, showed the most pronounced and highest antiplasmodial activity amongst all the other fractions with a low IC_50_ value of 1.25 μg/mL. CSE showed an IC_50_ value of 5.65 µg/mL, followed by the alkaloid fraction at 14.96 μg/mL. Tannin and saponin fractions showed IC_50_ values of 110.49 μg/mL and 128.74 μg/mL, respectively. The protein fraction showed no detectable antimalarial activity. The IC_50_ of reference standard artemether (ARM) was found to be 0.0343 μg/mL.

### 2.5. In Vivo Antimalarial Activity in P. berghei Infected Mice

In vivo efficacy of CSE and seed fractions was determined against *P. berghei* by monitoring percent parasitemia on the 7th and 14th days post-treatment, and the mortality of the mice was monitored on a daily basis. In the infected control group, which was receiving distilled water on a daily basis, all six mice were dead between the 7th and 14th days post-infection. The percent parasitemia of the infected control was found to increase from 54.18 to 97.43%, which was the highest observed among all the groups.

Each of the groups treated with the CSE and the Annomaal had 80% and 90% survival rates, respectively, on the 14th day, which was statistically significant (*p* < 0.05 compared to the infected control). Alkaloid and protein fractions showed 30% and 20% survival rates, respectively, whereas animals treated with saponin, and tannin fractions showed 100% mortality at the end of 14 days. Artemether (ARM), the positive control group, had a 100% survival rate by the 14th day post-infection (Table 7).

Parasitemia decreased gradually in all groups, except the infected control group wherein the mice died between days 7 and 14 from the start of the treatment. Parasitemia decreased from 55.18% on day 0 to 49.67% on day 7 with the lowest parasitemia (32.39%) observed in the positive control group treated with ARM at the end of the 14th day. Treatment with CSE showed a decline in parasitemia from 53.38% (day 0) to 47.94% (day 7) and 40.46% (day 14), respectively. Annomaal-treated mice recovered from symptoms of malaria and displayed a reduction of parasitemia from the initial 53.79% to 49.16% on day 7 and 37.89% on day 14, respectively. This reduction in the percent of parasitemia in Annomaal-treated groups was significant in comparison to the parasitemia of the infected control (*p* < 0.05). In the case of saponin and tannin fraction-treated animal groups, there was a decrease in the percent of parasitemia; however, the decrease was not statistically significant compared to the infected control (Table 7).

Chemosuppression is inversely related to parasitemia. Annomaal induced high chemosuppression (61.11%) amongst all the fractions of seeds of *A. squamosa.* The CSE showed chemosuppression of 58.47%. (Table 7). ARM displayed the highest chemosuppression of 66.75% as determined on the 14th day post-infection.

### 2.6. Dose and Dosage Regimen Determination Study

To determine the appropriate dose and dosage regimen of the oily fraction, Annomaal was administered orally at three dose levels of 150, 200, and 250 mg/kg/day following three dosage regimens viz. for 7 days, 5 days, and 3 days for each of the doses, respectively. The mice receiving a 150 mg/kg daily dose of Annomaal for 3, 5, and 7 days showed survival times of 5, 15, and 18 days, respectively. Animals treated for 3 and 5 days died within 7–10 days of the study. Animals treated for 7 days continually showed a 50% reduction in parasitemia by the end of 12 days. However, the parasitemia increased after the treatment was stopped, leading to the death of all animals in the group (Table 8).

Mice receiving 200 mg/kg daily dose for 3, 5, and 7 days showed survival of up to 30 days for 3-day dosing and 40 days for 5- and 7-day dosing regimens. The time required for a 90% reduction in parasitemia was 28 and 32 days, respectively, for animals treated for 7 and 5 days continually (Table 8). However, animals treated for 3 days only showed an initial decline in % parasitemia with 64.86 ± 1.39% by day 12, followed by a rapid increase leading to mortality of all the test animals by day 30. Mice receiving a 250 mg/kg daily dose for 3, 5, and 7 days showed survival of up to 40 days. The animals showed a 90% reduction in parasitemia at 32, 26, and 20 days, respectively.

### 2.7. GC-MS Analysis

GC-MS analysis shows that the oily fraction Annomaal consists of a mixture of various fatty acids expressed as methyl esters (Table 9). C-18 fatty acids linoleic and oleic acid were found to be the major components. Linoleic acid was found to be present in the highest concentration (38.86 %) followed by oleic acid (24.53%) and palmitic acid (17.08%) (Figures S1 and S2—Supplementary Materials).

## 3. Discussion

This study reports the scientific basis for the use of the seeds obtained from mature fruits of *A. squamosa* for its antimalarial activity. The phytochemical investigations showed that the powdered seeds of *A. squamosa* had a maximum yield of alkaloids (76.0%) followed by oils (20.98%) while the presence of saponin, tannins, and protein fractions were low. In vitro hemolysis tests were carried out to evaluate the membrane-rupturing properties of the CSE and the five fractions towards RBCs. The release of hemoglobin from the RBCs was used to determine if CSE or the seed fractions may have any damaging effect on RBCs membranes. Alkaloid and saponin fractions revealed some membrane-rupturing properties with the release of hemoglobin; however, the CSE, tannins, oil, and protein fractions were found to be safe, with the release of hemoglobin well within the tolerated levels of 5%. An acute in vivo oral toxicity study as per the OECD guidelines demonstrated the CSE and all the fractions to be of category 5 with no toxicity at the high dose of 2000 mg/kg.

Annomaal, the oily fraction obtained from powdered seeds of *A. squamosa*, showed the most pronounced and highest antiplasmodial activity amongst all the other fractions with a low IC_50_ value of 1.25 μg/mL. The inhibitory activity against *P. falciparum*, shown by the oily fraction, was higher than that reported for *A. squamosa* stem bark (IC_50_ of 8.5 μg/mL) and *A. squamosa* leaves extract with IC_50_ of 2 and 30 μg/mL for the chloroquine-sensitive and -resistant strains [28] and IC_50_ of 2.1 and 3.3 μg/mL in chloroquine-sensitive and -resistant strains as reported by Singh et al. [11]. According to the guidelines of the World Health Organization, extracts with IC_50_ ≤ 5 μg/mL are classified as having high or pronounced antimalarial activity [35], thus Annomaal has been demonstrated to be a promising candidate and was further evaluated in vivo for antimalarial efficacy.

*Plasmodium berghei* is very widely used in research programs for the development and screening of anti-malarial drugs, being one of the malaria parasites that infect mammals other than humans. These parasites have proven to be comparable to human malaria in most vital aspects of their structure, physiology, and life cycle, hence used for in vivo evaluation [36,37]. Annomaal-treated mice displayed the maximum reduction of parasitemia from 53.79% (day 0) to 49.16% (day 7) and 37.89% (day 14), respectively. Annomaal induced the highest chemosuppression (61.11%) amongst all the fractions of seeds of *A. squamosa,* hence confirming its antimalarial activity. A further attempt was made to ascertain the dosage regimen for Annomaal, which can enable a full recovery from the infection with total eradication of the parasite as resurgence in malaria is a major issue leading to drug resistance. In herbal practice, drugs are usually used in the form of infusions or decoctions with no major thrust on the dose and dosage regimen of the drug used [38,39]. The drug is usually taken until the point the symptoms of the ailment are eliminated. However, with the development of the modern system of medicine, it becomes essential for the standardization of dose and dosage regimen to ensure complete recovery, not only in terms of symptoms but also in terms of complete parasiticidal activity. It is essential to ascertain the dosage regimen, which can enable a full recovery from the infection with total eradication of the parasite, as the resurgence in malaria is a major issue leading to drug resistance. Three doses of 150 mg/kg, 200 mg/kg, and 250 mg/kg were evaluated. Peter‘s four-day test was modified wherein the treatment period was varied for 3, 5, and 7 days. The time taken for a 90% reduction in parasitemia, and survival rate of animals was observed. Once a daily dose of 250 mg/kg for 3 continuous days was found to be the optimum treatment regime for Annomaal as it not only decreased parasitemia but also led to the full survival of animals, which remained in good health. Though 3-day dosing of 250 mg/kg required a longer time for the reduction in parasitemia, no mortality of animals was observed even after 40 days of study. Prolonging the dosage regimen for 5 and 7 days did not show any betterment in terms of survival time. Thus, this dosage regimen would also permit patient compliance as it is once daily and limited to three days, unlike some of the other antimalarials, which may require frequent dosing and treatment for prolonged periods.

GCMS analysis confirmed that linoleic acid (38.86 %) and oleic acid (24.53%), the two C-18 fatty acids, as the major components present in Annomaal in line with previous reports [40,41]. Fatty acid biosynthesis is vital for parasite growth as fatty acids are essential for the development of cell membranes, are key sources of energy, play a crucial role in signal transduction as well as in protein acylation, and are required for growth, differentiation, and homeostasis in *P. falciparum*. Lipid biosynthesis is known to intensify during the erythrocytic phases of the parasite [42]. Interestingly, the *P. falciparum* parasite uses type II synthase while humans make use of type I synthase [43]. This provides an opportunity to selectively target the parasite’s fatty acid synthase without causing any damage to the host. The antimalarial effect of fatty acids has been realized; fatty acids themselves might impede the fatty acid biosynthesis of *P. falciparum,* presenting it as a possible strategy to defy the parasite [44]. The polyunsaturated fatty acids docosahexaenoic acid eicosapentaenoic acid, arachidonic acid, and linoleic acid caused a marked inhibition of in vitro growth of Plasmodium falciparum [45]. Kumarartilake [45] reported the activity of unsaturated fatty acids to increase with an increase in the degree of unsaturation. Krugliak et al. demonstrated the role of C18 fatty acids in the inhibition of *P. falciparum* in vitro and *P. vinckei* and *P. yoelli* nigeriensis in vivo. They reported the IC50 of linoleic acid to be 76 ± 3.7 μg/mL against *P. falciparum*, 23 ± 0.5 μg/mL for oleic acid, and 92 ± 1.8 μg/mL for linolenic acid [46]. Melariri et al. reported antiplasmodial activity <10 μg/mL for linoleic and linolenic acids and their methyl esters using a PLDH assay and the synergistic effect in vivo in a *P. berghei* model when provided in combination [47]. The presence of C18 fatty acids including linoleic acid, oleic acid, and their methyl esters would have contributed to the antiplasmodial effect of Annomaal. Annomaal was found to be more potent (IC50 value 1.25 μg/mL) than linoleic, oleic, or linolenic acids alone, which could be postulated due to the synergistic effect as it comprises a mixture of fatty acids and their methyl esters. The oxidation of methyl esters to their active metabolites would justify their in vivo efficacy when given orally. Fatty acid esters are metabolized as other dietary fats. They are hydrolyzed to the free fatty acids during digestion in the gut. Annomaal also showed higher antiplasmodial activity against *P. falciparum* than scleropyric acid, a C18 fatty acid isolated from the shoots of Scleropyrum plant (IC50 = 7.2 μg/mL) [48].

Lipid peroxidation has been suggested as the likely mechanism responsible for the antiplasmodial activity displayed by the n-3 and n-6 polyunsaturated fatty acids (PUFAs) [45]. Linoleic acid, present in Annomaal, is a ω-6 polyunsaturated fatty acid. When the PUFAs are incorporated into the parasites’ phospholipids or incorporated into the membranes of the infected cell, the oxidation radical produced by the parasite could enhance their peroxidation with damage to membrane integrity [45]. Another study reported that n-3 polyenoic fatty acids, eicosapentaenoic and docosahexaenoic acids, and the n-6 fatty acid arachidonic acid significantly enhanced neutrophil-mediated antiparasitic activity. [49]. However, fatty acids do not decrease cellular potassium, which could be detrimental to cell functions such as DNA synthesis or pyruvate kinase activity. Moreover, the fatty acid inhibitory effects were not mediated by depriving the parasite of essential nutrients or by preventing the exit of waste products [50]. They could inhibit the biosynthesis of type II fatty acid synthase, which takes place in the apicoplast of *P. falciparum* [43].

## 4. Materials and Methods

### 4.1. Plant Material

The seeds were sourced from mature fruits obtained from *A. squamosa* trees grown in Rajiv Gandhi National Park, Mumbai (geographical coordinates—longitude 720° 53′ to 720°58′ E, latitude 190°08′ to 190°21′ N) during the rainy months (June to September). Taxonomical identification of the specimen including leaves and flowering parts was carried out by a botanist from the Botanical Survey of India, Pune. Dried specimens were deposited at the herbarium of the Botanical Survey of India, Pune with the allocated voucher number of SS1/2008.

### 4.2. Preparation of the Plant Materials

The seeds were carefully removed from the pulp of the fruit. Any seeds with visible signs of bacterial or fungal infection were rejected. The seeds were thoroughly washed with purified water and allowed to dry in the air. Dried seeds were ground to a powder using a powder grinder. The powder (5 kg) was screened through 60 mesh to obtain a homogeneous powder, which was stored in dry conditions and used for all investigations.

### 4.3. Animals

Female Wistar albino rats, weighing 150–180 g, were used for acute toxicity studies. For in vivo four-day Peter’s suppression test for malaria and dosage regimen studies, Swiss albino mice of either sex, weighing 18–20 g, were used. All the study animals were housed in polypropylene cages under a standard light/dark cycle, with food and water given ad libitum. The experiments were performed between 8.00 and 16.00 h. The experimental protocols were approved by the Institutional Animal Ethics Committee of C. U. Shah College of Pharmacy, SNDT Women’s University, Mumbai, and conducted according to the guidelines of the Committee for the purpose of Control and Supervision of Experiments on Animals (CPCSEA), New Delhi, India.

### 4.4. Treatment of Blood

Whole human blood with the blood group of O positive was obtained from the Rotary Club Blood Bank, Mumbai. Citrate phosphate dextrose (CPD) buffer was used as an anticoagulant. The red blood cells (RBCs) pellet was acquired by spinning the blood for a period of 5 min at a g-force of 1200 and separating the plasma. The pellet was suspended in a washing medium constituting of the Roswell Park Memorial Institute (RPMI) 1640 medium added at a concentration of 10.4 g/L, 4-(2-hydroxyethyl)-1-piperazineethanesulfonic acid (HEPES −25 mM), gentamicin (12.5 μg/mL), and sodium bicarbonate (23.8 mM) with pH adjusted to 7.2. This was centrifuged for 10 min at 1200× *g*-force and the supernatant was removed. The washing step was carried out twice and, finally, the washed RBCs were suspended in the RPMI complete medium comprised of the washing medium (as above) with supplementation of 10% of serum inactivated by heating (30 min at 57 °C). The washed RBCs were kept at 4 °C and used to maintain the plasmodium culture (Section 4.5). Any unused RBCs were discarded after 4 days.

### 4.5. Parasite Culture

Cryopreserved parasite cultures of the *P. falciparum* 3D7 strain (chloroquine and Sulfadoxine/pyrimethamine resistant) were collected at the National Institute of Malaria Research, New Delhi repository. The glycerol stock of the parasite was rapidly thawed in a water bath at 37 °C for 1–2 min, and the sodium chloride solution was added gradually via swirling. The supernatant was discarded after centrifugation, and the parasite pellet was suspended in RPMI medium 1640 supplemented with HEPES (20 mmol/L) and gentamicin sulphate (50 mg/mL). Prepared RBCs (Section 4.4) were added at 5% hematocrit and transferred to a T25 cm^2^ culture flask and incubated at 37 °C under an atmosphere of 5% oxygen, 5% carbon dioxide, and 90% nitrogen. Media was changed daily to maintain the culture at parasitemia of 2 to 4%. Sorbitol (5%) was used to obtain synchronized cultures at 3% hematocrit.

### 4.6. Evaluation of Phyto-Constituents

Phytochemical analysis of the powdered seed and/or hydroalcoholic seed extract was performed in accordance with standard methods [51]. All analysis was performed in triplicates. The hydroalcoholic seed extract was prepared using 1:1: alcohol:water by the Soxhlet extraction method. The seed extract was hydrolyzed by boiling in a water bath with 1N HCl solution for 10 min. After cooling, the excess acid was neutralized using calcium carbonate until effervescence ceased and filtered. This extract was used for further tests to identify the presence of carbohydrates, proteins, fats, and oils, saponins, flavonoids, alkaloids and tannins, and phenolic compounds.

### 4.7. Preparation of Crude Extracts [52]

1. Preparation of crude seed extract (CSE): First, the seed powder was extracted with petroleum ether, and the petroleum ether layer was removed. The remaining solid mass was extracted using a 50:50 mix of water and alcohol in the Soxhlet extractor for a period of 24 h. The hydroalcoholic extract was collected and evaporated to remove the solvent. The dried crude seed extract (CSE) thus obtained was used for further investigations.
2. Preparation of alkaloid-rich fraction (F1): The seed powder was extracted with petroleum ether to remove the fat. Defatted powder mass was filled in the percolator, and the ammonia solution (10%) was passed for 24 h. The powder mass was then refluxed with methanol as an extraction solvent. The methanol layer was then removed by filtration and heated to obtain the concentrated alkaloid-rich fraction (F1). The extract was evaluated for phytochemical tests to confirm the presence of alkaloids.
3. Preparation of oily fraction (F2): Seed powder was extracted with petroleum ether in a Soxhlet extractor for 24 h. The petroleum ether layer was collected and heated carefully to evaporate the solvent and acquire the oil-rich fraction, which was labelled Annomaal (F2).
4. Preparation of saponin-rich (F3) and tannin-rich (F4) fractions: The seed powder was first extracted with petroleum ether to defat, and the defatted powder mass was then extracted with distilled water. The water layer was kept aside, and the plant material was further extracted with n-butanol. The N-butanol layer was separated by filtration and evaporated to obtain the saponin-rich fraction (F3), while the water layer was heated to dryness to acquire the tannin-rich fraction (F4).
5. Preparation of protein fraction (F5): The seed powder was defatted by extracting it with petroleum ether. Defatted powder mass was percolated with tris buffer saline in cold conditions (4 ± 2 °C) for a period of 24 h. The tris buffer saline layer was collected, and 90% ammonium sulphate was added to precipitate the proteins. The second precipitation was carried out with 50% ammonium sulphate. The precipitates were pooled and filled in a dialysis bag (150 kDa molecular weight cut-off) and dialyzed for 24 h with cold distilled water maintained at 4 ± 2 °C to remove any impurities.

### 4.8. GC–MS Analysis of the Oily Fraction—Annomaal

The oil of *Annona squamosa* (Annomaal) was analyzed using GC-MS analysis on the Jeol, Accu TOF GCV, Agilent 7980 model with FID detector. Compounds were separated on 30 m × 0.25 mm i.d. DB-17 capillary column. Prior to analysis, the sample containing fatty acids was esterified to the more volatile methyl esters by the methanol·BF3 method [53]. To optimize the separation of compounds, the elution temperature was programmed for a gradient of 2 °C/min from the initial temperature of 120 °C to 190 °C where it was held for 8 min. The carrier gas was nitrogen with a column flow rate of 1 mL/minute. To avoid column overload, the split mode was used at a ratio of 100:1, and the temperatures of the injector and detector were maintained at 250 °C.

### 4.9. In Vitro Erythrocyte Toxicity Test

This test evaluates the membrane-rupturing properties of the compounds towards RBCs. Heparinized blood collected from Wistar rats was used for the study. The RBC pellet obtained upon centrifugation of the blood was washed and suspended in cold phosphate buffer saline (PBS) pH 7.4. The prepared RBCs were incubated at 37 °C with CSE and five fractions of the *A. squamosa* seeds at a concentration of 250 mg/mL in PBS (pH 7.4) for sixty minutes. After incubation, the debris was separated by centrifugation and the supernatant was assayed for the released hemoglobin by estimating the absorbance at 540 nm on a UV spectrophotometer (UV 2450, Shimadzu, Kyoto, Japan). Triton X-100 (0.2% in PBS) was used as a positive control [54]. Hemolysis of less than 5% was considered to be at a safe level.

### 4.10. In Vivo Acute Oral Toxicity Study

An acute oral toxicity study was performed on the CSE and the five fractions of F1, F2, F3, F4, and F5 as per OECD guidelines [55] on female Wistar rats weighing 150–180 g. The animals were divided into 6 groups of six animals each. Each of the test animals was given a single dose of 2000 mg/kg body weight orally of the test samples (CSE or either of the five fractions suspended using 0.1% sodium carboxy methyl cellulose). All the animals were observed for changes in animal behavior, locomotor changes, feeding habits, and changes in body weight over the period of fourteen days.

### 4.11. In Vitro Anti-Plasmodial Efficacy Testing in P. falciparum Infected RBCs

An in vitro susceptibility assay was carried out using synchronized cultures of *P. falciparum* (3D7 strain) at the ring stage using the Trager Jensen method [29]. Synchronization was carried out using sorbitol lysis (5%). For antiplasmodial activity, 200 μL of the *P. falciparum* culture (5% hematocrit and 1.5% parasitemia) was seeded in a 24-well plate and CSE and five fractions of *A. squamosa* seeds micronized in RPMI 1640 medium were added at a dose range of 500, 250, 125, 62.5, 13.25, 6.12, 3.06, and 1.02 µg/mL. The plates were incubated at 37 °C for 48 h and parasitemia was estimated as the percentage of parasitemia after counting 1000 RBCs from each slide prepared from blood smears, after being fixed in methanol and stained with Giemsa stain (1:10 in Sorenson’s Buffer, pH 7.2). ARM was used as the standard, and the mean parasitemia inhibition was determined against infected drug-free control and IC_50_ values were calculated.

### 4.12. In Vivo Determination of Antimalarial Activity

In vivo antimalarial activity was determined by Peter’s 4-day suppressive antimalarial assay using the *Plasmodium berghei* culture acquired from Mumbai Veterinary College, Parel, Mumbai—400 012 [30]. The parasite culture was maintained in live mice as a host. Either sex Swiss albino mice (age—8 weeks, weight—18–20 g) were used for the experiments. Experimental malaria was induced by intraperitoneal injection of parasite inoculum (0.2 mL of blood with 1% parasitemia containing approximately 1.0 × 10^7^ parasitized RBCs/mL in isotonic saline) collected from donor mice. The infected mice were divided into eight groups. A completely randomized design was employed when conducting the experiment. The cages were assigned treatments randomly. The treatment was initiated four hours after the infection. Mice received oral administration of each of the test samples (CSE and the five fractions suspended using 0.1% sodium carboxy methyl cellulose) at a dose of 250 mg/kg/day (2 mg/mouse/day) for 4 days (Group I–VI); there was also one positive control (Group VII) and one negative control (Group VIII). Artemether at the dose of 40 mg/kg/day (0.2 mg/mouse/day) was used as a positive control while the negative control group was treated with distilled water (0.2 mL/mouse/day).

Blood smears were prepared (days 4, 7, and 14) for each of the experimental mice by collecting blood by giving a prick in the tail. Methanol was used for fixing the smears and staining was performed with the Geimsa stain (10%). The slides were observed at 100× oil immersion lens under a compound microscope and the total number of RBCs and number of parasitized RBCs were determined per magnification field. Observations were recorded from four magnification fields and 1000 RBCs were counted from each slide and the percent of parasitemia was calculated. The data obtained were used to determine the percentage of chemosuppression in each mouse using Equation (1). The antimalarial activity was reported using equation 2 as per the standard protocol [56].

% Chemosuppression = [(A − B)/A] × 100 = %(1)

where A is the mean parasitemia in the negative control group and B is the mean parasitemia in test groups.

% Antimalarial Activity = 100 − (Mean parasitemia of treated group/Mean parasitemia of control group) × 100(2)

The number of dead mice was also recorded each day since the start of the 4-day suppressive test for the next 30 days.

### 4.13. Dose and Dosage Regimen Determination Study

Malaria-infected mice were randomly segregated into 9 groups of five animals each group. The mice were assigned to random treatments. The study was performed by modifying Peter’s four-day test. The oily fraction, Annomaal, was administered orally at three dose levels of 150, 200, and 250 mg/kg/day following three dosage regimens (7 days, 5 days, and 3 days) for each of the doses. The time required for a reduction in the percent of parasitemia by 90% of induced parasitemia was noted and the survival time was observed.

### 4.14. Statistical Analysis

One-way ANOVA followed by Tukey’s multiple comparison test was used to evaluate the data expressed as Mean ± S.E. Graphpad instat Demo version was used for the statistical analysis. *p*-value < 0.05 was considered significant.

## 5. Conclusions

Several studies have been conducted to present the scientific basis for the use of plants for ethnomedicinal use against malaria; however, this study reports for the first time the in vitro and in vivo antimalarial activity of the seeds of *A. squamosa* and its phytoconstituents. In addition, this study validates the dose and dosage regimen required to obtain a complete parasiticidal effect leading to the complete cure of malaria in infected mice. The study showed that the CSE, protein, saponin, and oily fractions obtained from the seeds of *A. squamosa* were nontoxic at 2000 mg/kg body weight dose with high safety and classified as class 5. CSE, tannin, oily, and protein fractions showed acceptable hemolytic activity less than 5%. The oily fraction Annomaal showed pronounced antiplasmodial activity with a low IC_50_ value (1.25 ± 0.183 μg/mL) against *P. falciparum*. The CSE and Annomaal significantly inhibited the growth of *P. berghei* parasites in vivo. Annomaal showed high chemosuppression of 61.11% indicating good antimalarial activity against *P. berghei* parasites. Furthermore, the study has shown that oral administration of Annomaal at a daily dose of 250 mg/kg/day for 3 consecutive days was sufficient for the complete cure of *P. berghei*-infected mice. The suggested dosage regimen would be patient-compliant as it requires only once-daily dosing for a short treatment period of three days, eliminating the need for frequent dosing for a prolonged period of time as required by several synthetic antimalarial drugs. Annomaal has been shown to be rich in fatty acids, with a linoleic acid content of 38.86%, which could play an important role in the inhibitory action against the parasite. Further studies using bioassay-guided fractionation of Annomaal are needed to isolate and identify the active compounds responsible for the antimalarial activity. Their active constituents may be potential candidates with therapeutic value in the treatment of malaria in the future.

## Figures and Tables

**Table 1:** Phytochemical analysis of the seed powder of *Annona squamosa*.

Test	Result	References
Test for Carbohydrates		[32]
1. Molisch test	+	
2. Fehling’s test	+	
3. Benedict’s test	+	
4. Barfoed’s test	-	
5. Bial’s test	-	
6. Selwinoff’s test	-	
**Test for Proteins**		[32]
1. Biuret test	+	
2. Million’s test	+	
3. Precipitation test	+	
4. Test for proteins containing Sulphur	-	
5. Xanthoprotein test	-	
**Test for Fats and Oils**		[32]
1. Solubility test	+	
2. Filter paper test	+	
3. Saponification test	+	
4. Salkowski test for steroids	+	
**Test for Flavonoids**		[32]
1. Shinoda test	+	
2. Lead acetate test	+	
**Test for Alkaloids**		[32]
1. Dragendorff’s test	+	
2. Mayer’s test	+	
3. Hager’s test	+	
4. Wagner’s test	+	
5. Murexide test for purine alkaloids	-	
**Test for Saponins**		[32]
1. Foam test	+	
**Test for Tannins and Phenolic compounds**		[32]
1. 5% FeCl_3_ test	+	
2. Dilute Iodine test	+	
5. Acetic acid test	+	

+ means present, - means absent.

**Table 2:** Percent yield of the various phytoconstituents obtained from the seeds of *Annona squamosa*.

Phytoconstituent	Appearance	Yield (% *w*/*w*)
Crude Seed Extract	Brown sticky extract	75.29–79.93
Alkaloids	Brown powder	72.55–76.00
Oils and Fats	Light yellow oil	17.96–20.98
Saponins	Brown sticky mass	5.49–5.50
Tannins	Brown sticky mass	2.26–3.00
Proteins	Light buff powder	6.20–6.50

**Table 3:** Percentage hemolysis of rat red blood cells with CSE of *Annona squamosa* and its various fractions of seed extract (*n* = 3).

Fraction	Test Substance	Percent Hemolysis (Mean ± SD)
	Crude Seed extract (CSE)	4.89 ± 0.29
F1	Alkaloid fraction	11.57 ± 0.85
F2	Oil fraction	2.25 ± 0.19
F3	Saponin fraction	8.22± 0.39
F4	Tannin fraction	3.27± 0.23
F5	Protein fraction	1.73 ± 0.15

**Table 4:** Comparative weights of Wistar rats (*n* = 6) on oral administration of CSE of *Annona squamosa* and its various fractions at doses of 2000 mg/kg of body weight.

	Weight of Animals (g) ± SEM		
Test Substance	Day 0	Day 7	Day 14
Crude seed extract (CSE)	156.7 ± 1.3	159.7 ± 4.4	161.24 ± 1.39
Alkaloid fraction	153.5 ± 0.27	129.6 ± 1.26	130.72 ± 1.93
Saponin fraction	154.02 ± 1.58	155.2 ± 0.32	157.5 ± 2.13
Oil fraction	153.2 ± 2.67	154.9 ± 1.04	156.5 ± 2.47
Tannin fraction	153.27 ± 1.05	154.94 ± 1.5	156.83 ± 1.79
Protein fraction	162.49 ± 1.21	163.5 ± 1.24	164.19 ± 2.48

*p* < 0.05, SEM—Standard Error of Mean.

**Table 5:** Cage-side observations for Wistar rats (*n* = 6) treated with CSE and seed fractions of *Annona squamosa*, after acute oral toxicity study.

Observation	Crude Seed Extract	Alkaloid Fraction	Saponin Fraction	Oily Fraction	Protein Fraction	Tannin Fraction
Changes in fur	0/6	0/6	0/6	0/6	0/6	0/6
Eyes and mucous membranes	0/6	0/6	0/6	0/6	0/6	0/6
Respiratory abnormality	0/6	0/6	0/6	0/6	0/6	0/6
Circulatory	0/6	0/6	0/6	0/6	0/6	0/6
Autonomic	0/6	0/6	0/6	0/6	0/6	0/6
Somatomotor Activity	0/6	0/6	0/6	0/6	0/6	0/6
Behaviour abnormality	0/6	0/6	0/6	0/6	0/6	0/6
Locomotion	0/6	3/6	0/6	0/6	0/6	0/6
Inactivity	0/6	3/6	0/6	0/6	0/6	0/6

**Table 6:** Antiplasmodial activity of CSE of *Annona squamosa* and its various fractions on *P. falciparum*.

Fraction	Test Substance	IC_50_ (μg/mL) ± SEM
	Crude Seed extract (CSE)	5.65 ± 0.279
F1	Alkaloid fraction	14.96 ± 0.352
F2	Oil fraction	1.25 ± 0.183
F3	Saponin fraction	128.74± 0.239
F4	Tannin fraction	110.49± 0.173
F5	Protein fraction	-

*p* < 0.05, SEM—Standard Error of Mean (*n* = 3).

**Table 7:** Mean parasite density, chemosuppression, and survival time of *Plasmodium berghei*-infected mice post-treatment with CSE and various fractions of *Annona squamosa*.

Group	Phytoconstituent Fraction	Mean (X ± SEM) Parasite Density on Day 0 (%) (% Parasitemia)	Mean (X ± SEM) Parasite Density on Day 7 (%) (% Parasitemia)	Mean (X ± SEM) Parasite Density on Day 14 (%) (% Parasitemia)	Mean (X ± SEM) Chemosuppression on Day 14 (%)	Mice Survival by Day 14 (%)
I	Crude extract (CSE)	53.38 ± 0.67	47.94 ± 1.59	40.46 ± 2.29	58.47 ± 1.36	80
II	Oil fraction, Annomaal	53.79 ± 1.05	49.16 ± 1.46	37.89 ± 1.52	61.11 ± 2.59	90
III	Alkaloid fraction	54.98 ± 1.95	59.34 ± 2.63	62.25 ± 3.40	36.10 ± 2.94	30
IV	Protein fraction	53.17 ± 1.02	61.59 ± 2.95	80.37 ± 4.78	17.51 ± 2.48	20
V	Saponin fraction	54.26 ± 0.93	71.45 ± 1.30	92.29 ± 2.96	5.27 ± 3.19	0
VI	Tannins fraction	54.96 ± 1.60	68.28 ± 2.73	90.10 ± 1.18	3.70 ± 2.46	0
VII	Std ARM	55.18 ± 0.92	49.67 ± 1.05	32.39 ± 2.30	66.75 ± 1.69	100
VIII	Infected Control	54.18 ± 0.40	97.43 ± 1.64	-	0	0

*p* < 0.05, SEM—Standard Error of Mean.

**Table 8:** Survival time and time required for 90% reduction in parasitemia at various dose levels and dosing periods with Annomaal in *Plasmodium berghei*-infected mice.

Dose (mg/kg/day)	150	200	250	150	200	250
Dosing Period (Days)	Survival Time (Days)			Time Required (Days) for 90% Reduction in Parasitemia		
7	18	40	40	NC	28	20
5	15	40	40	NC	32	26
3	5	30	40	NC	NC	32

NC (not calculated)—Not calculated as the animals showed mortality before 90% reduction in parasitaemia could be achieved.

**Table 9:** Components of oily fraction Annomaal as obtained by GC-MS analysis.

Peak No.	Retention Time (min.)	Peak Area (%)	Constituent Present	Molecular Formula	Molecular Weight
1	12.11	17.08	Palmitic acid methyl ester	C_14_H_34_O_2_	270.5
2	12.94	24.53	Oleic acid methyl ester	C_19_H_36_O_2_	296.5
3	13.12	38.86	Linoleic Acid methyl ester	C_19_H_34_O_2_	294.5
4	14.96	11.27	Palmitoleic acid methyl ester	C_17_H_32_O_2_	268.4
5	15.22	2.36	Myristic acid methyl ester	C_15_H_30_O_2_	242.4

## Data Availability

All data analyzed during this study are included in this article.

## Supplementary Materials

The following supporting information can be downloaded at: https://www.mdpi.com/article/10.3390/molecules28145472/s1, Figure S1. GC-MS Chromatogram of Annomaal showing peaks of various fatty acid methyl esters with linoleic acid methyl ester peak shown at retention time of 13.12 mins. Figure S2. Mass spectra of 9,12-0ctadecedienoic acid, methyl ester (Linoleic Acid methyl ester).

## Sample Availability

Samples of the Annomaal oil fraction can be made available from the authors.
