# Supplementary material for: In Vitro Effect on Plasmodium falciparum and In Vivo Effect on Plasmodium berghei of Annomaal, an Oily Fraction Obtained from the Seeds of Annona squamosa
Source: Molecules. 2023 Jul 17;28(14):5472. doi: 10.3390/molecules28145472 (PMC10383673; doi:10.3390/molecules28145472)
Supplement: Supplementary file 1 [file molecules-28-05472-s001.zip › molecules-2294143-supplementary.pdf]

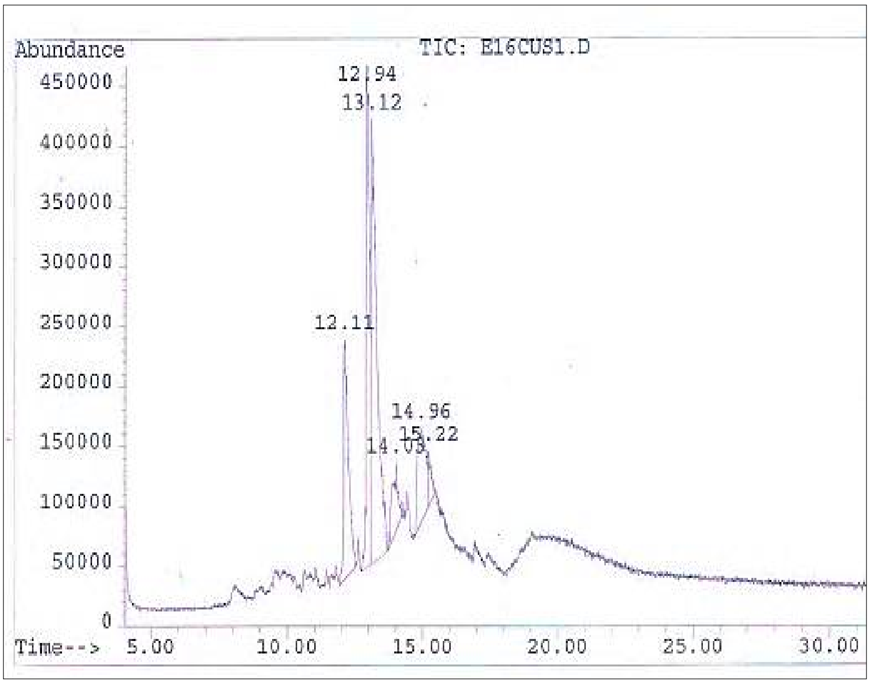


**Figure S1.** GC-MS Chromatogram of Annomaal showing peaks of various fatty acid methyl esters with linoleic acid methyl ester (major component) peak shown at retention time of 13.12 mins.

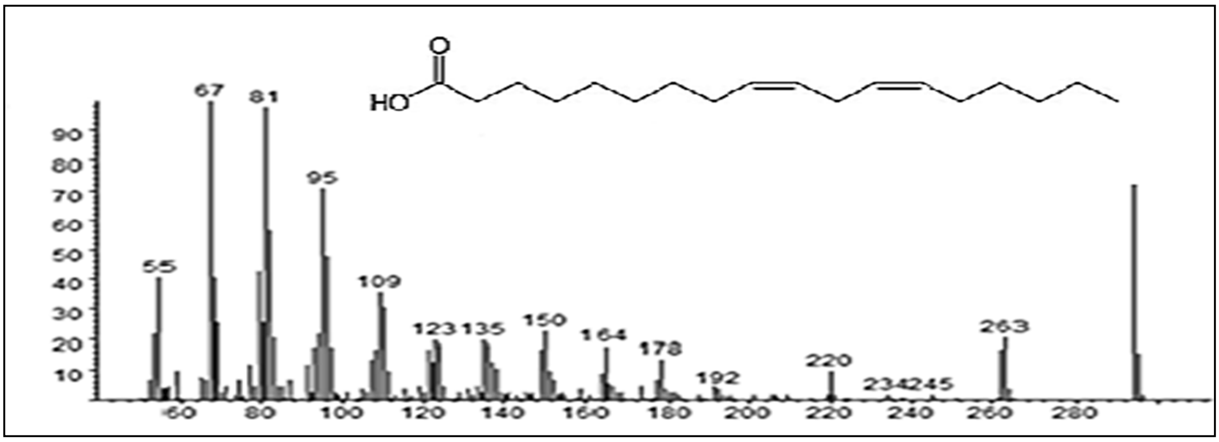


**Figure S2.** Mass spectra of 9,12-0ctadecedienoic acid, methyl ester (Linoleic Acid methyl ester).
